# Cemented Versus Uncemented Total Hip Arthroplasty in Sickle Cell Disease Patients: A Retrospective Study

**DOI:** 10.7759/cureus.36138

**Published:** 2023-03-14

**Authors:** Osama Almarzooq, Mohamed Alhassan, Layla Alansari, Turki Alanazi, Fatema H Madan

**Affiliations:** 1 Orthopaedics and Trauma, Salmaniya Medical Complex, Manama, BHR; 2 Orthopaedic Surgery, Salmaniya Medical Complex, Manama, BHR

**Keywords:** cemented total hip arthroplasty, cementless total hip arthroplasty, hip joint pain, avascular necrosis (avn), sickle cell disease

## Abstract

Background and objective

Sickle cell disease (SCD) can predispose patients to avascular necrosis (AVN) of the femoral head, resulting in severe disabling pain. Total hip arthroplasty (THA) is the leading treatment choice for end-stage arthritis caused by AVN. In this study, we aimed to compare complications associated with implant fixation with and without cement.

Materials and methods

We retrospectively analyzed 95 total hip implants in which 26 patients had staged bilateral THA. These surgeries were performed by four senior arthroplasty consultants between 2007 and 2018. Data were collected from the surgical logbook, physical files, and the electronic patient database (I-Seha, National Health Information System, Ministry of Health, Kingdom of Bahrain).

Results

The study included 95 hip implants in 69 patients. Forty-five (47%) were in males, and 50 (53%) were in females. Of these, 22 implants underwent revision (23%), two implants had periprosthetic infections (0.2%), two implants had periprosthetic fractures (0.2%), and 18 implants had implant loosening. We found that cemented THA was significantly associated with the development of implant loosening (p<0.001), small particle disease (p<0.001), and a higher revision rate (p<0.001).

Conclusion

We found that cemented THA in SCD patients led to a higher rate of aseptic implant loosening, mainly caused by osteolysis. Based on our findings, we recommend uncemented THA in SCD patients.

## Introduction

Sickle cell disease (SCD) is considered one of the most significant hereditary blood disorders due to its chronic nature and associated high morbidity and mortality. The prevalence of SCD in Bahrain was reported to be 2.1% in a previous neonatal screening study conducted from 1984 to 1985, which is considered high [1]. SCD results from a point mutation in the β-globin chain of hemoglobin replacing the amino acid glutamate with valine at the sixth position. The association of two normal α-globin subunits with two mutant β-globin subunits forms hemoglobin S [2]. This results in hyperplasia of bone marrow, sickling of red blood cells, increased blood viscosity, and eventually arterial occlusion and venous obstruction [3].

Avascular necrosis (AVN) of the femoral head is one of the most disabling sequelae of SCD, with femoral head collapse, arthritis, and debilitating hip pain. The prevalence of AVN in SCD ranges from 10 to 40% [4-6]. Many of these patients are initially asymptomatic. Unmanaged asymptomatic femoral head osteonecrosis in SCD patients has a high risk of advancing to pain and collapse. It has been reported that 95% of the asymptomatic patients with normal radiographs and abnormal MRI became symptomatic with hip pain within three years [7].

Many treatment modalities have been described to treat AVN in various stages of arthritis, including bisphosphonates in the early degenerative phase, as well as operative management with core decompression, rotational osteotomy, and total hip arthroplasty (THA). THA is the leading treatment choice for end-stage arthritis. Due to the young and active nature of patients affected with AVN, high implant-related complications have been reported, mainly due to these patients’ high functional demands [8].

The method of fixation of primary total hip implants is still a matter of controversy. Some prefer using cementing as a method of fixation, while others believe that the uncemented method leads to better functional outcomes and fewer implant-related complications. No definitive piece of evidence has been proposed to establish that one of these methods is superior to the other [9,10]. This paper aims to compare the complications of cemented versus uncemented THA in SCD patients.

## Materials and methods

Study design and setting

We retrospectively analyzed 95 THAs in 69 patients. Twenty-six of the patients had staged bilateral THAs. Patients were operated on between 2007 and 2018 by four senior arthroplasty consultants at the Salmaniya Medical Complex, the largest public hospital in Bahrain. All the patients were diagnosed with SCD with advanced AVN of the femur head.

Data were collected from the surgical logbook, physical files, and the electronic patient database (I-Seha, National Health Information System, Ministry of Health, Kingdom of Bahrain). Any short-term or long-term follow-up, such as for superficial or deep tissue infection, deep venous thrombosis, implant failures such as implant loosening, and the need for revision, was also documented. Patients who underwent THA for conditions unrelated to SCD were excluded.

Surgical methods and implant choice

All patients had undergone the procedure using the posterior approach to the hip. All patients had metal on highly cross-linked polyethylene bearings. There was no hybrid fixation in the study population. The use of drains was decided based on the surgeons' preference.

Diagnosing complications

Implant loosening was diagnosed with a plain radiograph and CT scan of the affected hip joint. Physical and electronic files were reviewed to investigate other complications such as periprosthetic infections, periprosthetic fractures, or thromboembolic events.

Statistical analysis

Analysis was conducted using IBM SPSS Statistics version 28 (IBM Corp., Armonk, NY). To summarize the data, descriptive statistics were utilized, and the frequencies and proportions were calculated for the demographic and clinical characteristics of the study population. Furthermore, a Chi-square test of independence was performed to analyze the relationship between the type of fixation and complications and to analyze risk factors predisposing to revision surgery.

## Results

The study included a total of 95 hip implants in 69 patients. Forty-five (47%) were in males, and 50 (53%) were in females. Twenty-six patients had staged bilateral THAs. Fifty-five (58%) implants used uncemented fixation, and 40 implants had cemented fixation (42%). A total of 22 implants underwent revision (23%), two (2%) implants had periprosthetic infections, two (2%) had periprosthetic fractures, and 18 implants had implant loosening (Table 1). A total of 22 (23.16%) implants had evidence of implant loosening on the CT scan of the hip joint, four (4.21%) implants had femoral component loosening, five (5.26%) implants had acetabular cup loosening, and 13 (13.68%) implants experienced loosening of both components. Eighteen (18.95%) implants with implant loosening underwent revision surgery, but four did not.

**Table 1:** Demographic and clinical characteristics of the study population (n=95 implants, 69 patients) Note: Hₐ is proportion ≠ 0.5

Variable	Level	Number of implants	Proportion
Gender	Male	45	0.47
	Female	50	0.53
Cemented/uncemented	Uncemented	55	0.58
	Cemented	40	0.42
Periprosthetic infection	No	93	0.98
	Yes	2	0.02
Revision	No	73	0.77
	Yes	22	0.23
Osteolysis	No	78	0.82
	Yes	17	0.18
Component loosening	No	73	0.77
	Yes	22	0.23

We found a statically significant association between cemented THA and the development of implant loosening (p<0.001), small particle disease (p<0.001), and a higher revision rate (p<0.001) (Table 2).

**Table 2:** Comparison between the rate of complications in cemented and uncemented hip replacement *Statistically significant (p<0.05)

Variable	Level		Uncemented	Cemented	χ²	P-value
Periprosthetic fracture	No	Observed	55	38	2.81	0.094
		Expected	53.84	39.16		
	Yes	Observed	0	2		
		Expected	1.16	0.84		
Revision	No	Observed	49	24	11.01	<0.001*
		Expected	42.26	30.74		
	Yes	Observed	6	16		
		Expected	12.74	9.26		
Osteolysis	No	Observed	53	25	18.07	<0.001*
		Expected	45.16	32.84		
	Yes	Observed	2	15		
		Expected	9.84	7.16		
Component loosening	No	Observed	49	24	11.01	<0.001*
		Expected	42.26	30.74		
	Yes	Observed	6	16		
		Expected	12.74	9.26		
Small particle disease	No	Observed	55	35	7.26	0.007*
		Expected	52.11	37.89		
	Yes	Observed	0	5		
		Expected	2.89	2.11		
Dislocation	No	Observed	54	40	0.74	0.391
		Expected	54.42	39.58		
	Yes	Observed	1	0		
		Expected	0.58	0.42		
Thromboembolic event	No	Observed	55	39	1.39	0.238
		Expected	54.42	39.58		
	Yes	Observed	0	1		
		Expected	0.58	0.42		

Furthermore, we investigated factors leading to revision surgery and found it to be statistically significantly associated with cemented fixation (p<0.001), osteolysis (p<0.001), and component loosening (p<0.001) (Table 3).

**Table 3:** Analysis of the factors predisposing to revision surgery *Statistically significant (p<0.05)

Variable	Level		Revision		χ²	P-value
			No	Yes		
Gender	Male	Observed	31	14	3.04	0.081
		Expected	34.58	10.42		
	Female	Observed	42	8		
		Expected	38.42	11.58		
Periprosthetic infection	No	Observed	73	20	6.78	0.009*
		Expected	71.46	21.54		
	Yes	Observed	0	2		
		Expected	1.54	0.46		
Periprosthetic fracture	No	Observed	73	20	6.78	0.009*
		Expected	71.46	21.54		
	Yes	Observed	0	2		
		Expected	1.54	0.46		
Cemented	No	Observed	49	6	11.01	<0.001*
		Expected	42.26	12.74		
	Yes	Observed	24	16		
		Expected	30.74	9.26		
Osteolysis	No	Observed	69	9	33.07	<0.001*
		Expected	59.94	18.06		
	Yes	Observed	4	13		
		Expected	13.06	3.94		
Component lessening	No	Observed	69	4	55.36	<0.001*
		Expected	56.09	16.91		
	Yes	Observed	4	18		
		Expected	16.91	5.09		
Small particle disease	No	Observed	72	18	9.58	<0.001*
		Expected	69.16	20.84		
	Yes	Observed	1	4		
		Expected	3.84	1.16		
Dislocation	No	Observed	73	21	3.35	0.067
		Expected	72.23	21.77		
	Yes	Observed	0	1		
		Expected	0.77	0.23		
Thromboembolic events	No	Observed	73	21	3.35	0.067
		Expected	72.23	21.77		
	Yes	Observed	0	1		
		Expected	0.77	0.23		

One (1.05%) implant had deep venous thromboembolism, and no implants had heterotopic ossifications or intraoperative periprosthetic fracture. Eighty-six implants had a drain placed intraoperatively, and only nine (9.47%) implants did not have a drain placed.

## Discussion

Fixation of hip implants can be classified into either cemented fixation using polymethylmethacrylate or biologic uncemented fixation relying on bone ingrowth and ongrowth. Several studies have concluded that cementless hip arthroplasty is superior to the cemented method. A couple of papers have cited results that prove that cemented hip arthroplasties lead to more complications than cementless ones [11-13]. With trends leaning toward the biological fixation of hip implants, this study aimed to investigate the complications associated with the method of fixation of total hip implants in SCD patients.

Unlike osteoarthritis (OA) patients who receive THA, patients with SCD have elevated risks of complications following this procedure. A retrospective study involving 881 patients found a higher rate of aseptic loosening (1.94%) in patients with SCD compared to patients with AVN without SCD (0.68%; p=0.021) [14].

Good outcomes were reported following uncemented THAs in a study by Ilyas et al., which reported 133 uncemented THAs with a mean follow-up of 14.6 years and a 94.1% survival rate at 15 years [15]. Azam and Sadat-Ali assessed 87 cementless procedures and reported a survival rate of 92.6% at 7.5 years [16]. Although these two papers followed only uncemented fixation, their results are in agreement with our findings in terms of higher implant survival in uncemented patients.

Several papers have described various concepts with regard to THA in SCD patients, including the systematic review conducted by Kenanidis et al. Their study's findings are in agreement with ours, revealing a higher rate of revision in cemented THA (48 out of 312 cemented THAs were revised at a mean of 13 years versus 14 out of 133 uncemented THAs at a mean of 14.6 years) [17]. Similarly, AlOmran compared cemented versus uncemented THAs and found that 61% (p=0.001) of the cemented implants failed compared to 22.3% of the uncemented implants [12].

This study has a few limitations, primarily related to lost details and data; moreover, its retrospective design proved to be an obstacle in documenting the functional status of the patients pre-and postoperatively. Additionally, the two groups of patients were operated on by different surgeons, and factors such as surgical techniques may have influenced the results. Finally, it was not documented if different implant brands were used, and this may have been a confounding factor.

## Conclusions

THA is the preferred management of choice for patients with advanced AVN secondary to SCD. The primary goal of this procedure is to alleviate hip pain and improve the range of motion of the hip joint. However, elevated complications associated with SCD might affect the quality of life following this procedure; hence, eliminating factors related to these complications might aid in improving outcomes postoperatively. We found that cemented THA in SCD patients led to a higher rate of aseptic implant loosening, mainly caused by osteolysis. Furthermore, cemented fixation technique resulted in a higher rate of revision surgery. In light of our results, we recommend the use of uncemented fixation for total hip implants in SCD patients.
